# A novel RNA modification prognostic signature for predicting the characteristics of the tumor microenvironment in gastric cancer

**DOI:** 10.3389/fonc.2023.905139

**Published:** 2023-02-16

**Authors:** Qiangan Jing, Hongfeng Yao, Huanjuan Li, Chen Yuan, Jiayu Hu, Ping Zhang, Yunyi Wu, Yi Zhou, Xueying Ren, Chen Yang, Guojie Lei, Jing Du, Xia Ke, Jun Xia, Xiangmin Tong

**Affiliations:** ^1^ College of Biotechnology and Bioengineering, Zhejiang University of Technology, Hangzhou, Zhejiang, China; ^2^ Laboratory Medicine Center, Department of Clinical Laboratory, Zhejiang Provincial People’s Hospital, Affiliated People’s Hospital, Hangzhou Medical College, Hangzhou, Zhejiang, China; ^3^ Department of Clinical Laboratory, Zhuji People’s Hospital of Zhejiang Province, Zhuji, Zhejiang, China

**Keywords:** gastric cancer, RNA modification, tumor microenvironment, tumor mutational burden, immunotherapy

## Abstract

Gastric cancer (GC) is one of the most common neoplastic malignancies, which permutes a fourth of cancer-related mortality globally. RNA modification plays a significant role in tumorigenesis, the underlying molecular mechanism of how different RNA modifications directly affect the tumor microenvironment (TME) in GC is unclear. Here, we profiled the genetic and transcriptional alterations of RNA modification genes (RMGs) in GC samples from The Cancer Genome Atlas (TCGA) and Gene Expression Omnibus (GEO) cohorts. Through the unsupervised clustering algorithm, we identified three distinct RNA modification clusters and found that they participate in different biological pathways and starkly correlate with the clinicopathological characteristics, immune cell infiltration, and prognosis of GC patients. Subsequently, univariate Cox regression analysis unveiled 298 of 684 subtype-related differentially expressed genes (DEGs) are tightly interwoven to prognosis. In addition, we conducted the principal component analysis to develop the RM_Score system, which was used to quantify and predict the prognostic value of RNA modification in GC. Our analysis indicated that patients with high RM_Score were characterized by higher tumor mutational burden, mutation frequency, and microsatellite instability which were more susceptible to immunotherapy and had a favorable prognosis. Altogether, our study uncovered RNA modification signatures that may have a potential role in the TME and prediction of clinicopathological characteristics. Identification of these RNA modifications may provide a new understanding of immunotherapy strategies for gastric cancer.

## 1 Introduction

Gastric cancer (GC) is one of the most prevalent cancers and the third leading cause of cancer-related deaths, with a variety of factors that ordinarily facilitate its occurrence (1, 2). Although therapeutic approaches in radiotherapy and chemotherapy are improving clinically, the myriad of GC patients who are diagnosed in the advanced stage do not benefit from it (3). Consequently, in clinical practice, improving the sensitivity of early diagnostic tools and treatment with effective drugs are the most useful strategies for increasing the survival rate. Previous studies have demonstrated that changes in structural genomics have an inextricable correlation with cancers, and certain genetic changes (e.g., mutation and duplication) may have a perceptible effect on the progression of GC patients (4). Accumulating studies have revealed that DNA copy number variations (CNV) have a significant role in gene expression and tumorigenesis of GC, acting as a crucial influential factor on oncogenic pathways (5, 6).

Post-transcriptional modification is an essential regulatory section in the progression of many diseases and ubiquitously exists in virtually all cellular RNAs (7, 8). As yet, more than 150 different types of post-transcriptional RNA modifications have been found (9). N6-methyl adenosine (m6A) methylation is a newly recognized epigenetic modification and one of the most abundant forms of RNA modifications in eukaryotic cells. The formation and removal of m6A modification were a sort of dynamic reversible processes, which were implemented by methyltransferases known as “writers” (RBM15, ZC3H13, METTL3, METTL14, WTAP, and KIAA1429) and demethylases also called “erasers” (FTO and ALKBH5) (10). There is a growing body of literature supporting that m6A modification is involved in a series of bioprocesses regulations, including RNA translation, degradation, nuclear output, and disease states, especially in tumor malignant progression and immunomodulatory abnormalities (11–16). The N1-Methyladenosine (m1A) modification, present in a minority of mRNAs, is regulated by the enzymes known as”writers” of TRMT61A, TRMT61B, TRMT10C, and TRMT6. m1A modification usually plays a translational repression role that can have a devastating effect on base pairing, and also affects the tertiary structure of ribosomes through ribosomal scanning or translation (17, 18). The alternative polyadenylation (APA) is a ubiquitous RNA modification that can shear mRNA and adds poly(A) tails (19), it plays an essential role in regulating the stability and translation efficiency of target RNA. The synthesis of poly(A) are regulated by CPSF1, CPSF2, CPSF3, CPSF4, CSTF1, CSTF2, CSTF3 CFI, PCF11, CLP1, NUDT21, and PABPN1 protein complex (20). Additionally, adenosine-to-inosine (A-to-I) RNA editing, a crucial form of RNA modification, emerges in coding/non-coding regions of mRNAs and is mediated by adenosine deaminase, which frequently works on ADAR1 and ADAR2 (21, 22). Increasing research asserted that RNA modifications were tightly associated with human diseases, and the misregulation of RNA modification pathways may lead to cancer onset (7, 23). The interaction of different RNA modifications have not been fully elucidated. Therefore, we focused on the above four types of RNA modification to investigate the effect of distinct RNA modification patterns on gastric cancer.

Recently, immunotherapy has revolutionized the treatment strategies for multiple cancer, including gastric cancer. Although immunotherapy could elicit greater durable responses compared with conventional chemotherapy in advanced cancer patients, only a small number of patients can obtain positive responses and benefit from it (24). It is well known that immunotherapy responses were typically dependent on the immune cells inside the complex tumor microenvironment (TME). The TME, an integral part of tumor cells, can widely implicate tumorigenesis by regulating different signaling pathways (25). Growing studies have revealed the special crosstalk between RNA modification and the TME that the epigenetic modification of eukaryotic mRNA potentially relied on the TME infiltrating immune cells (26, 27). It has been reported that METTL3-mediated m6A methylation accelerated the activation and maturation of dendritic cells (28). The specific depletion of METTL3 resulted in the decreased expression of costimulatory molecules CD40 and CD80, impairing the capacity of stimulating T cell activation in turn (29). In addition, overexpression of METTL3 can facilitate GC malignant progression *via* angiogenesis and glycolysis signaling pathways (30). However, the limitation of technology contributed to most studies focusing on only one or two RNA modification regulators and cell types, whereas the antitumor effect is regulated by various tumor factors interacting under high coordination. Therefore, identifying the infiltration characterizations of TME immune cells mediated by RNA modification regulators will help us enhance insight into the immune regulation of TME in gastric cancer.

This study was designed to assess the expression profiles of 26 RMGs in 373 TCGA-STAD and 433 GEO samples of gastric cancer. We presented a comprehensive overview of RNA modification patterns and evaluated the relationship between those patterns and characteristics of TME immune cell infiltration. Interestingly, the TME characteristics of the three RNA modification patterns highly coincided with the immune-excluded, immune-desert, and immune-inflamed phenotypes (29). Moreover, we established a set of score schemes to quantify the RNA modification patterns in individual tumors and forecast the clinical characteristics and prognostic outcomes. Our study suggested that RNA modification plays a crucial role in the formation of the tumor immune microenvironment and is integral in the development of therapeutic intervention plans for patients with GC.

## 2 Materials and methods

### 2.1 Data sources and pro-processing

Public gene expression data (fragments per kilobase million, FPKM), genome mutation data, copy number variation data, and complete clinical annotations of GC, containing 343 tumor patients and 30 normal control samples, were derived from TCGA database (https://portal.gdc.cancer.gov/). The gastric adenocarcinoma datasets GSE84437 (433 tumor samples), GSE63089 (90 samples), and GSE27342 (160 samples) were downloaded from the GEO database (https://www.ncbi.nlm.nih.gov/geo/). Since the sample size of GSE84437 is greater than GSE63089 and GSE27342, we used GSE84437 for initial analysis to improve the accuracy and reliability of the analysis, and datasets GSE63089 and GSE27342 were used to validate the mRNA expression of hub genes. 26 RNA modification genes have been described in the study of Chen et al. The fpkm function of the “limma” package was performed to transform FPKM values of gene expression into transcripts per kilobase million (TPM) values (31), which was more in common with the data of the GEO chip. The “maftools” R package was used to draw the waterfall plots of RMGs mutation frequencies. The diagram of the location of RMGs alteration on 23 chromosomes was generated *via* the “RCircos” package.

### 2.2 Consensus clustering analysis of RNA modulators

The 26 RMGs, including 7 m6A modification genes (METTL3, METTL14, WTAP, RBM15, RBM15B, ZC3H13, VIRMA), 4 m1A modification genes (TRMT61A, TRMT10C, TRMT61B, TRMT6), 12 APA modification genes (CPSF1, CPSF2, CPSF3, CPSF4, CSTF1, CSTF2, CSTF3, CFI, PCF11, CLP1, NUDT21, PABPN1), and 3 A-I modification genes (ADAR, ADARB1, ADARB2). We used the unsupervised clustering analysis to determine distinct RNA modification patterns in gastric cancer based on the expression profiles of 26 RMGs and categorize patients for subsequent analysis. The consensus clustering algorithm was performed to determine the cluster number and stability (32). The “ConsensusClusterPlus” R package was utilized to implement the above steps and 1000 replicates were performed to ensure the stability of the categorization (33).

### 2.3 Clinical characteristics analysis based on different RNA modification patterns

To investigate the relationships between different RNA modification patterns and clinical characteristics, the clinical data (age, sex, TNM stage, survival status) was integrated into our study. The “c2.cp.kegg.v6.2 symbol” gene set that downloaded from the Molecular Signatures Database (MSigDB) (http://www.broad.mit.edu/gsea/msigdb/) (34) for running Gene Set Variation Analysis (GSVA) enrichment analysis, and “GSVA” R package was applied to identify the differences among three RNA modification patterns and biological characteristics. Adjusted *P*-value < 0.05 was viewed to be statistically significant. We used the Cox regression model to evaluate the survival prognostic of GC patients in different RNA modification patterns. The “survival” and “survminer” R packages were used for the generation of survival curves.

### 2.4 The TME cell infiltrating characteristics analysis

The gene set of TME infiltration immune cell has been described in the study of Charoentong et al. (35), which harbors numerous human immune cell subtypes such as activated CD8 T cell, activated dendritic cell, activated B cell, macrophage, mast cells, monocyte, natural killer T cell, and regulatory T cells. The relative abundance of each TME cell infiltration in each cluster was calculated by the single-sample gene-set enrichment analysis (ssGSEA) algorithm and represented by enrichment scores (29, 36).

### 2.5 Identification of DEGs and construction of RNA modification scoring system

We used the empirical Bayesian approach of the “limma” R package to identify DEGs among three RNA modification clusters under adjusted *P*-value < 0.001 conditions. Gene ontology (GO) functional annotation and Kyoto Encyclopedia of Genes and Genomes (KEGG) pathway enrichment analysis of those signature genes were conducted by using the “clusterProfler” R package with FDR < 0.01 condition, and then the results were visualized by the “ggplot2” R package. Next univariate Cox regression analysis was utilized to identify the marked prognostic-related genes between the clusters. Then, the principal component analysis (PCA) was used to build the RM_Score. Both principal components 1 and 2 were selected as signature scores, the advantage of this approach was that the score concentrated on the set with the largest blocks of highly correlated (or anti-correlated) genes in the set, while reducing the weight to genes that are not tracked with other set members. The RM_score is defined below:


$$RM\_Score=\Sigma (PC1_{i}+PC2_{i})$$

Where i is the expression of RNA modification phenotype-related genes. Finally, patients were classified into the high RM_Score group and low RM_Score group for deeper analysis according to the maximally selected rank statistics.

### 2.6 Correlation of the RM_Score and immune infiltration, tumor mutation burden, and microsatellite instability

We used the log-rank tests and Kaplan–Meier curves analysis to explore the prognostic value of the RM_Score. *P*-value < 0.001 was regarded to be statistically significant. Stratified analysis was used to determine whether the RM_Score maintained its predictive capacity in different groups. The ssGSEA algorithm was performed to estimate the infiltration of immune cells between the two distinct RM_Score groups. The Chi-square tests were applied to analyze the correlation of RM_Score and clinical characteristics. In addition, the Wilcoxon ranked-sum test was used to determine the differential expression of PD-1, and PD-L1 in the two RM_Score groups. Moreover, through the Spearman correlation analysis, the potential relationship between RM_Score and tumor mutational burden (TMB) was characterized as well as microsatellite instability (MSI).

### 2.7 Protein-protein interactions network construct and hub genes expression validation

We used the STRING database (https://cn.string-db.org/ ) to construct the protein-protein interactions (PPI) network of the 26 RMGs. The network was visualized by Cytoscape and the hub genes among 26 RMGs were screened by the cytoHubba plug-in of Cytoscape. The datasets of GSE63089 and GSE27342 were used for validation of the hub gene mRNA expression. The protein expression of hub genes in normal gastric and tumor tissue was investigated by employing The Human Protein Atlas (https://www.proteinatlas.org/).

### 2.8 Statistical analysis

The correlation coefficients between the infiltration of TME cells and the expression of RMGs were calculated *via* Spearman and distance correlation analyses. Wilcoxon rank-sum test and chi-square test were used to perform difference comparisons of continuous variables and classified variables between two groups, respectively. Difference comparisons among three or more groups were analyzed by One-way ANOVA and Kruskal-Wallis test (29, 37). The “survminer” R package was used for determining the optimum cut-off point for each dataset subgroup based on the relationship of RM_Score and survival in patients. On the basis of maximally selected log-rank statistics, the “surv-cutpoint” function was used to dichotomize RM_Score, GC patients were classified into the high RM_Score group and the low RM_Score group subsequently. For prognostic analysis, the Kaplan-Meier approach was carried out to generate survival curves and the significance of differences among groups was determined by log-rank tests. Univariate regression analyses were adopted to calculate the hazard ratios (HR) for RMGs and RNA modification signature genes. Identification of independent prognostic factors was applied through a multivariable Cox regression model. We used the “waterfall” function of the “maftools” R package to present the mutation landscape in patients with high and low RM_Score groups in the TCGA-STAD cohort. All statistical analyses were carried out with R 4.1.0 software. *P*-value < 0.05 was considered as statistical significance.

## 3 Results

### 3.1 Genetic and transcriptional alterations of RMGs in GC

**Figure 1:**
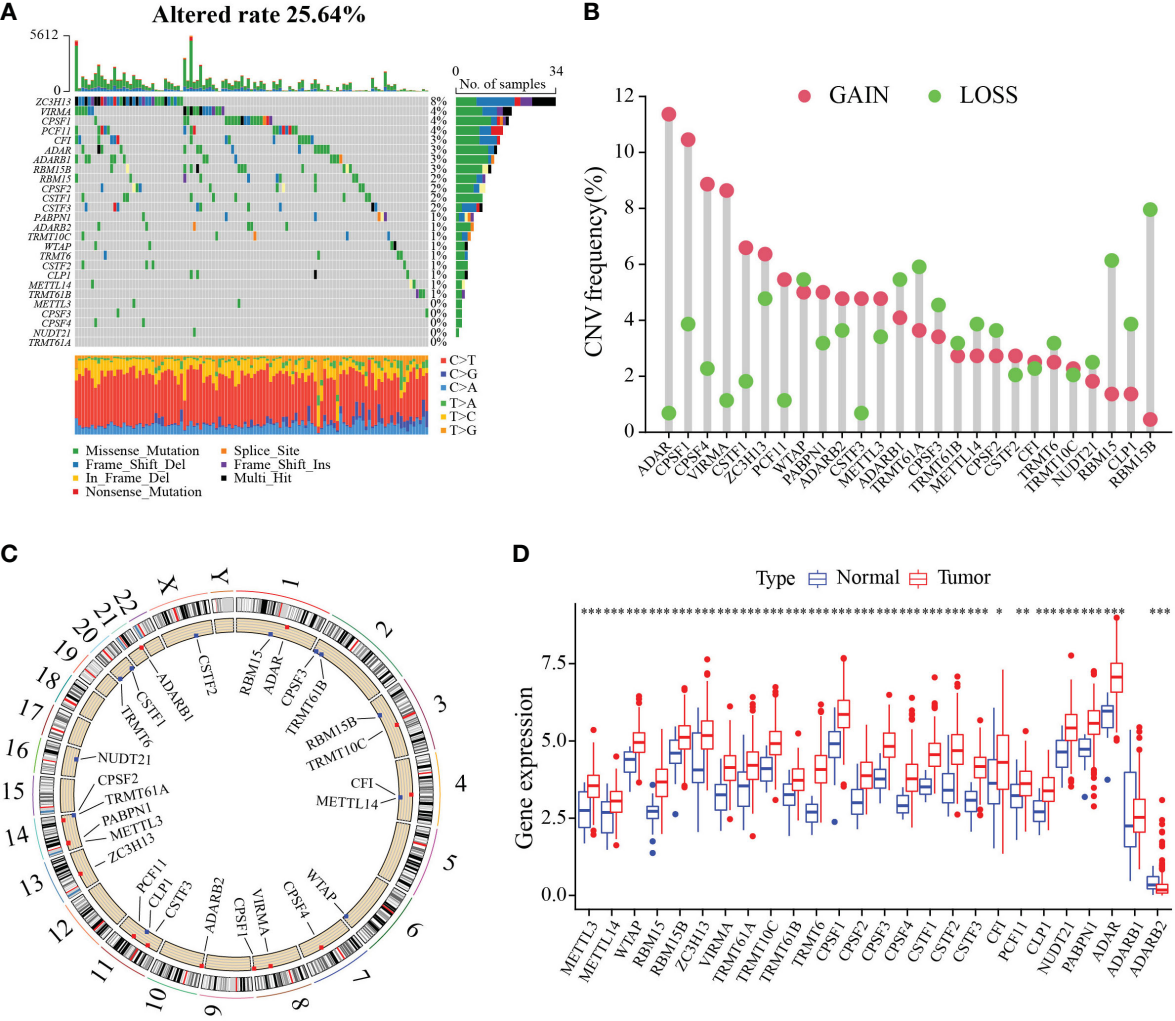
Genetic and transcriptional alterations of RNA modification regulators in gastric cancer. **(A)** Waterfall diagram of RMGs mutation frequency in TCGA-STAD cohort. **(B)** Histogram plot showing the CNV frequency of each gene based on statistical analysis among copy number of RMGs. Red dots indicate an increase in copy number, and green dots represent a reduction in copy number. **(C)** Circle diagram of the alterations sites of RNA modification-related genes CNV on 23 chromosomes. **(D)** Box plot displaying the expression distributions of RMGs between normal and tumor samples. Red or blue dots represent tumor and normal samples, respectively. *P-value < 0.05, **P-value < 0.01, ***P-value < 0.001.

To characterize the genetic and transcriptional changes of RMGs, we first analyzed the mutation frequencies of the 26 RMGs (**Table S1**). 25.64% of the GC samples in the TCGA cohort harbor gene alteration in these RMGs (**Figure 1A**). For that ZC3H13 harbors the highest mutation rate, we then divided the patients into two groups according to the ZC3H13 mutation profiles and found that METTL3 expression was significantly higher in the mutation group, whereas the expression of CFI and ADARB2 was remarkably increased in the wild group, suggesting ZC3H13 may be a regulator in mRNA expression (**Figure S1**). The somatic CNV of 26 RMGs was explored, and almost RMGs displayed CNV alterations (**Figure 1B**). The CNV alteration sites of RMGs on the chromosomes were shown in **Figure 1C**. We then assessed the RMGs expression between GC and normal tissues. Results revealed that the expression levels of almost all the RMGs were ubiquitously elevated in GC compared to normal tissues (**Figure 1D**). Furthermore, survival analysis showed that most of the RMGs have a strong correlation with the survival outcome of GC patients (**Figures S2A–D**). Collectively, these results unveiled the distinct characteristics of RMGs in both genetic alterations and transcriptional changes in GC and revealed that the up-regulated RMGs expression partly contributed to CNV alteration.

### 3.2 RMGs subtypes and clinicopathological analysis

**Figure 2:**
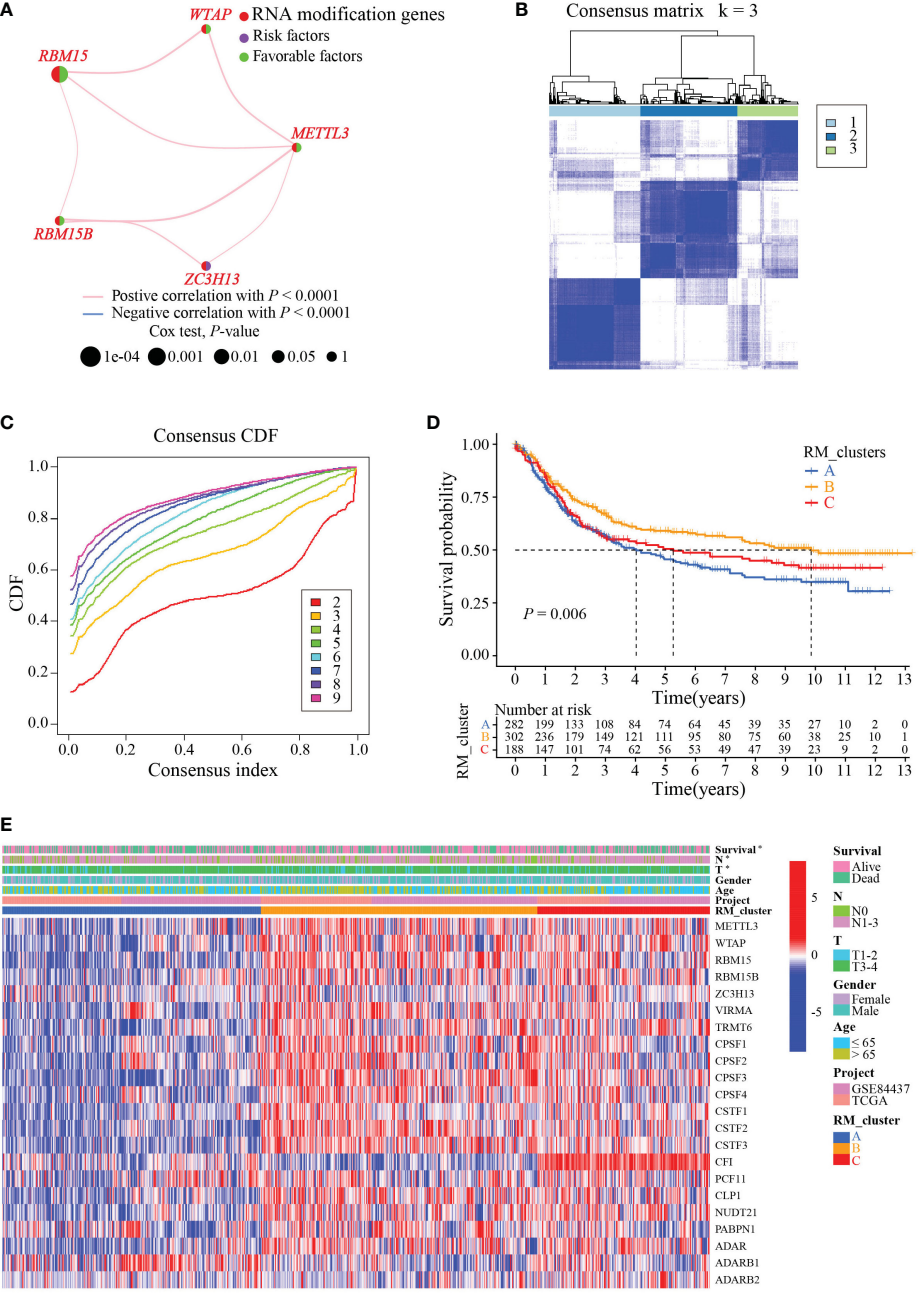
RMGs subtypes, clinicopathological and biological characteristics analysis. **(A)** Interaction networks diagram showing the positively correlated RNA modification genes in prognosis. The purple and green semicircles represent risk and favorable factors, respectively. **(B)** Consensus matrix heatmap of three clusters (k = 3) and their corresponding area. **(C)** The CDF curves when taking different k values. **(D)** Survival rate of gastric cancer patients in three RM_clusters based on univariate analysis. **(E)** Clinicopathologic characteristics and expression levels of RMGs among the three distinct RM_clusters. *P-value < 0.05.

**Figure 3:**
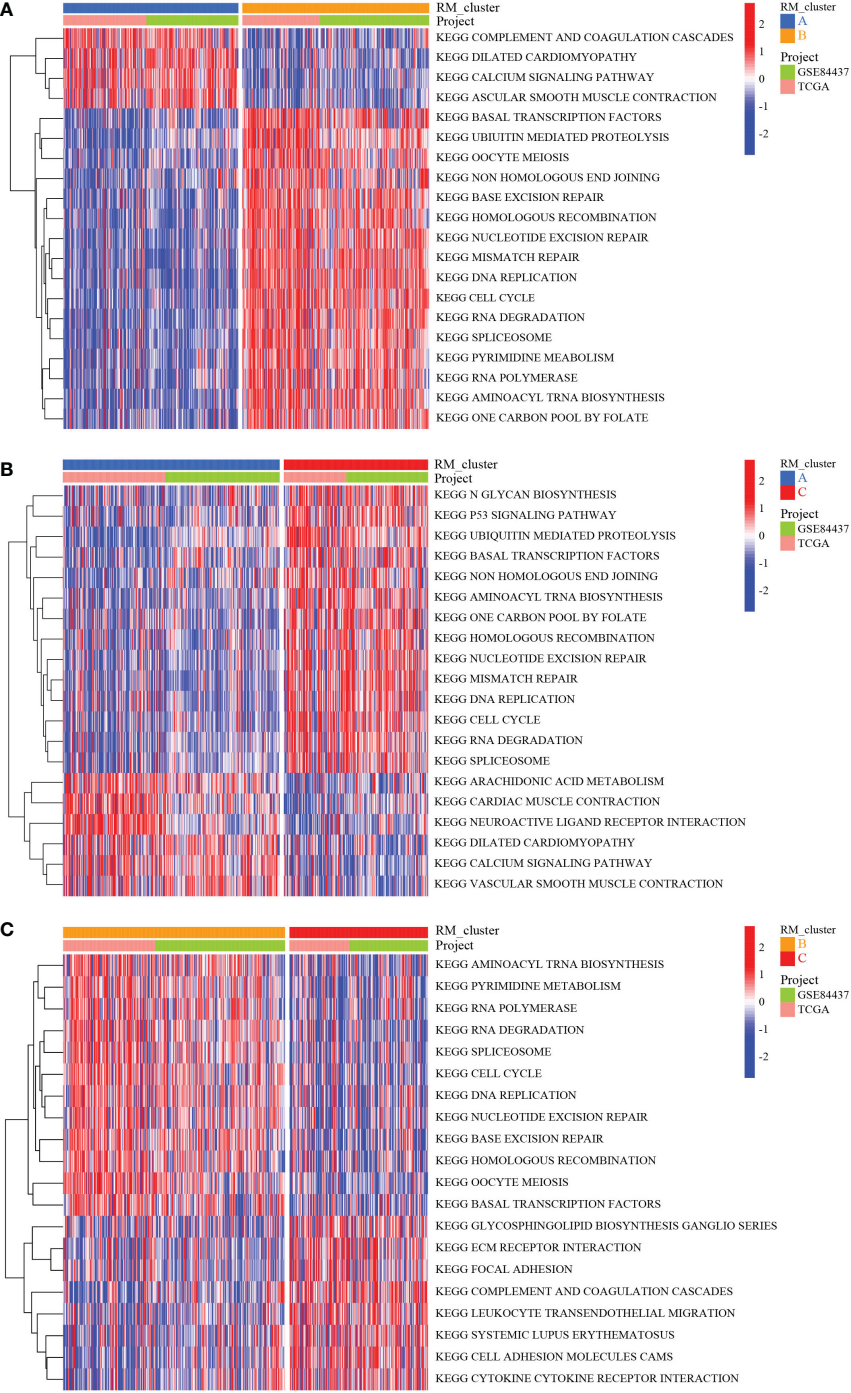
Biological characteristics analysis of three RNA modification clusters. **(A)** GSVA of the differences in biological pathways between RM_cluster A and B. **(B)** Differences in biological features between RM_cluster A and C. **(C)** Differences in biological features between RM_cluster B and C. Blue, orange, and red stripes represent three distinct RM_clusters.

To identify the subtypes of RMGs in GC, we integrated the data set GSE84437 and TCGA-GC in our study, and the Cox regression was used to reveal the survival prognostic values, and the prognosis interaction networks diagram based on multivariate Cox regression analysis suggested that five RMGs were positively correlated to each other (**Figure 2A**; **Table S2**). Subsequently, GC patients were categorized into different distinct RNA modification clusters, and the unsupervised clustering showed that k = 3 seemed to be the optimized choice (**Figures 2B, C**). The prognostic analysis showed that patients in RM_cluster B had a superior survival rate to those in RM_clusters A and C (**Figure 2D**). Heatmap indicated that the clinicopathologic characteristics of GC patients were discrepant strikingly, and the expression levels of most RMGs were markedly deficient in RM_cluster A and enriched in RM_cluster B (**Figure 2E**). Furthermore, GSVA enrichment analysis was performed to investigate the differences among three RNA modification patterns and biological characteristics. Notably, RM_cluster A was mainly concentrated in complement and coagulation cascades, dilated cardiomyopathy, and calcium signaling pathways; RM_cluster B was initially associated with pyrimidine metabolism, RNA polymerase, and oocyte meiosis signaling pathways; RM_cluster C was highly enriched in the immune-related pathways, including the complement and coagulation cascades, ECM receptor interaction, leukocyte transendothelial migration, and cytokine-cytokine receptor interaction signaling pathways (**Figures 3A–C**). These results suggested that the three categorized RMGs patterns had significantly different biological characteristics and could discriminate the prognosis of GC patients.

### 3.3 Characteristics of TME, and biological processes in three distinct RNA modification patterns

**Figure 4:**
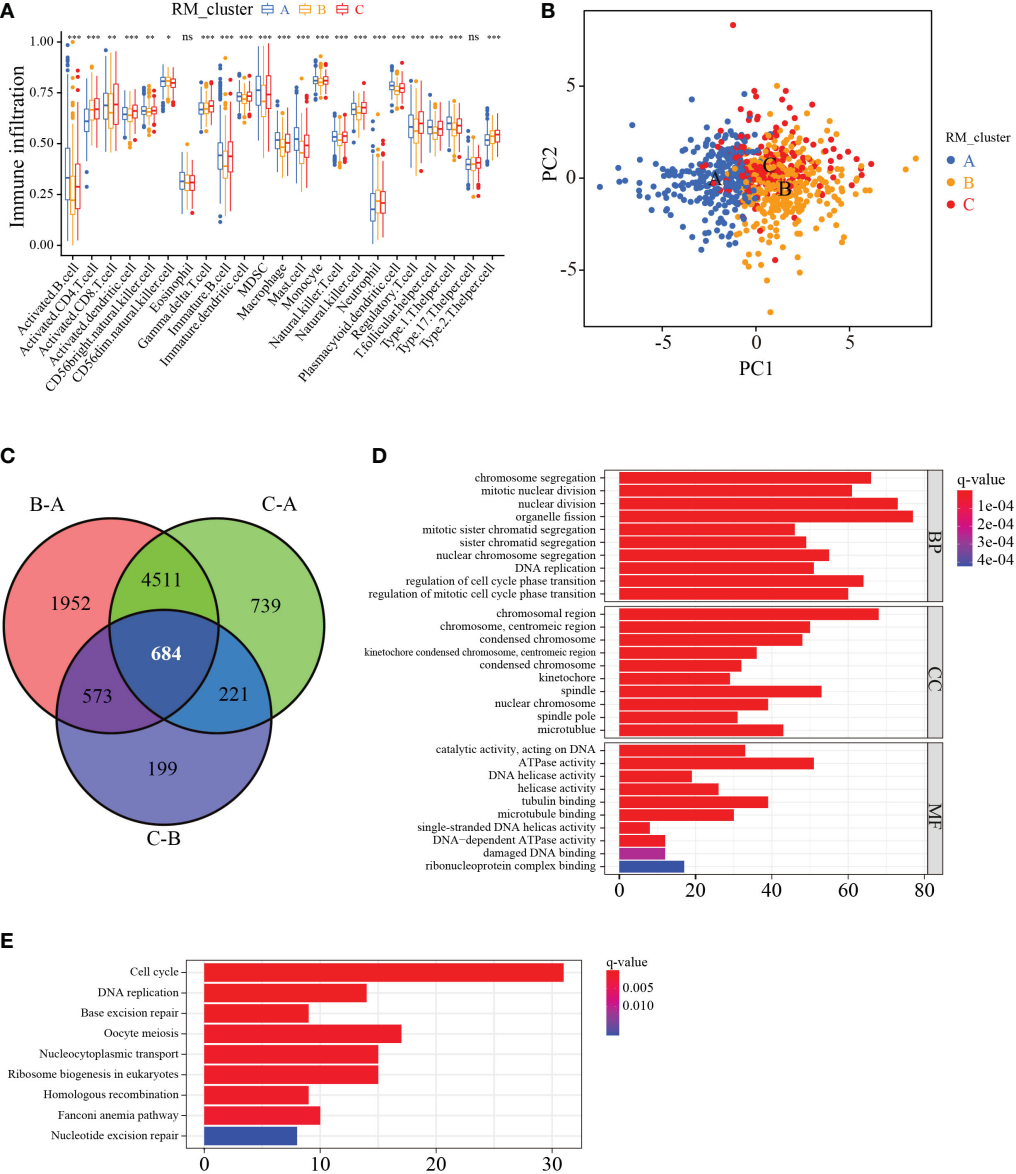
Characteristics of immune cell infiltration and function enrichment in three distinct RM_clusters. **(A)** Immune infiltration level of 23 immune cell types among the three RM_clusters. **(B)** Scatter plot of transcriptome features *via* PCA analysis. **(C)** Venn plots showing the overlapping genes in three RM_clusters. **(D)** GO enrichment analysis of overlapping genes. **(E)** KEGG enrichment analysis of overlapping genes. *P-value < 0.05, **P-value < 0.01, ***P-value < 0.001, ns represents no statistical significance.

The ssGSEA enrichment analysis was conducted to explore the infiltration characteristics of the immune cells among the three RNA modification patterns. The distribution of infiltration abundance of immune cells among three RNA modification patterns was significantly different (**Figure 4A**). PCA analysis showed the RNA modification profiles among the three distinct subtypes were markedly different (**Figure 4B**). To further investigate the potential biological characteristics of each RM_cluster, we confirmed 684 overlapping genes from the three RM_clusters based on these RNA modification-related DEGs (**Figure 4C**). After that, functional annotations of these overlapping genes were carried out by GO and KEGG enrichment analysis. The histogram of GO analysis showed that the 684 overlapping genes were mainly enriched in organelle fission, nuclear division, chromosomal region, spindle, ATPase activity, and tubulin binding (**Figure 4D**). The KEGG enrichment analysis indicated that these genes were major engaged in cell cycle, oocyte meiosis, nucleocytoplasmic transport, and ribosome biogenesis in eukaryotes (**Figure 4E**), indicating that RNA modification subtype-related genes play a significant role in cell division or growth.

### 3.4 Identification of RMGs signature and functional annotation

**Figure 5:**
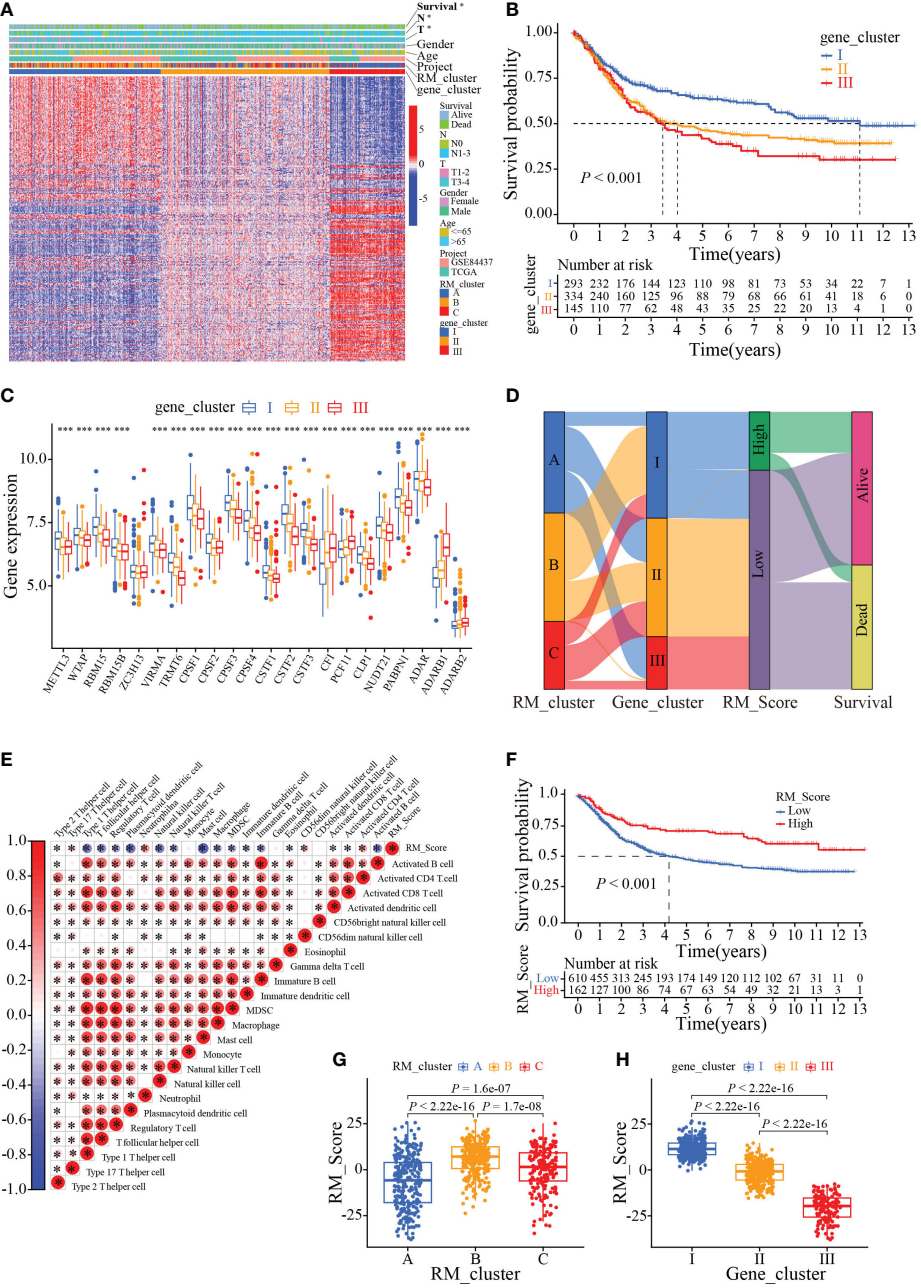
Phenotypic characteristics of the RMGs gene subtypes. **(A)** Relationships between clinicopathologic characteristics and genetic modification patterns. **(B)** Kaplan-Meier curves of the patient’s survival rate among different gene clusters. **(C)**. Expression levels of RMGs in different gene clusters. **(D)** Sankey diagrams of genotype distributions in three gene clusters and survival outcomes. **(E)** Correlation between RM_Score and multiple immune infiltrating cells. Red and blue represent positive or negative correlations, respectively, and * was considered statistically significant. **(F)** Kaplan-Meier analysis of survival probability of patients with gastric cancer in low or high RM_Score group. **(G)** The differences in RM_Scores among three RM_clusters. **(H)** The differences in RM_Scores among three gene clusters. *P-value < 0.05, ***P-value < 0.001.

To further ascertain the potential biological signature of the RMGs subtypes, 298 prognostic-related genes were identified from the three RM_clusters. Applying the unsupervised clustering algorithm, GC patients were classified into three genomic phenotypes (gene cluster I, gene cluster II, and gene cluster III) based on the 298 prognostic-related genes (**Figures S3A–L**). The heatmap of clinicopathologic characteristics and genetic modification patterns revealed that the expression abundance of the 298 genes was significantly reversed difference in gene cluster III and gene cluster I (**Figure 5A**). Survival analysis suggested that patients in gene cluster I had the best survival outcome, while patients in clusters III had the worst (**Figure 5B**). Additionally, the expression profiles of RMGs were significantly different among three gene clusters, which was parallel to the three RNA modification patterns results (**Figure 5C**). To quantify the three distinct RNA modification patterns in each GC patient, we established the RM_Score system, and GC patients were divided into RM_Score high and RM_Score low groups. The Sankey diagram showed the flow of the RM_Score and the last survival outcome of GC patients (**Figure 5D**). Correlation analysis of RM_Score and immune cells implied the T cell, and mast cell was tightly negatively correlated with RM_Score (**Figure 5E**). In addition, we found that RM_Score high patients had a better survival prognosis, which is in line with the Sankey diagram that most of the high score patients flow into the alive outcome (**Figure 5F**). Furthermore, the difference in RM_Score has been assessed in three RM_clusters and three gene clusters, which indicated that the highest RM_Score were in RM_cluster B and gene cluster I, respectively (**Figures 5G, H**). These results implied RM_Scores had a close correlation with the RMGs subtype and could be a suitable marker for predicting survival status.

### 3.5 Characteristics of RNA modifications in TMB and immune functions

**Figure 6:**
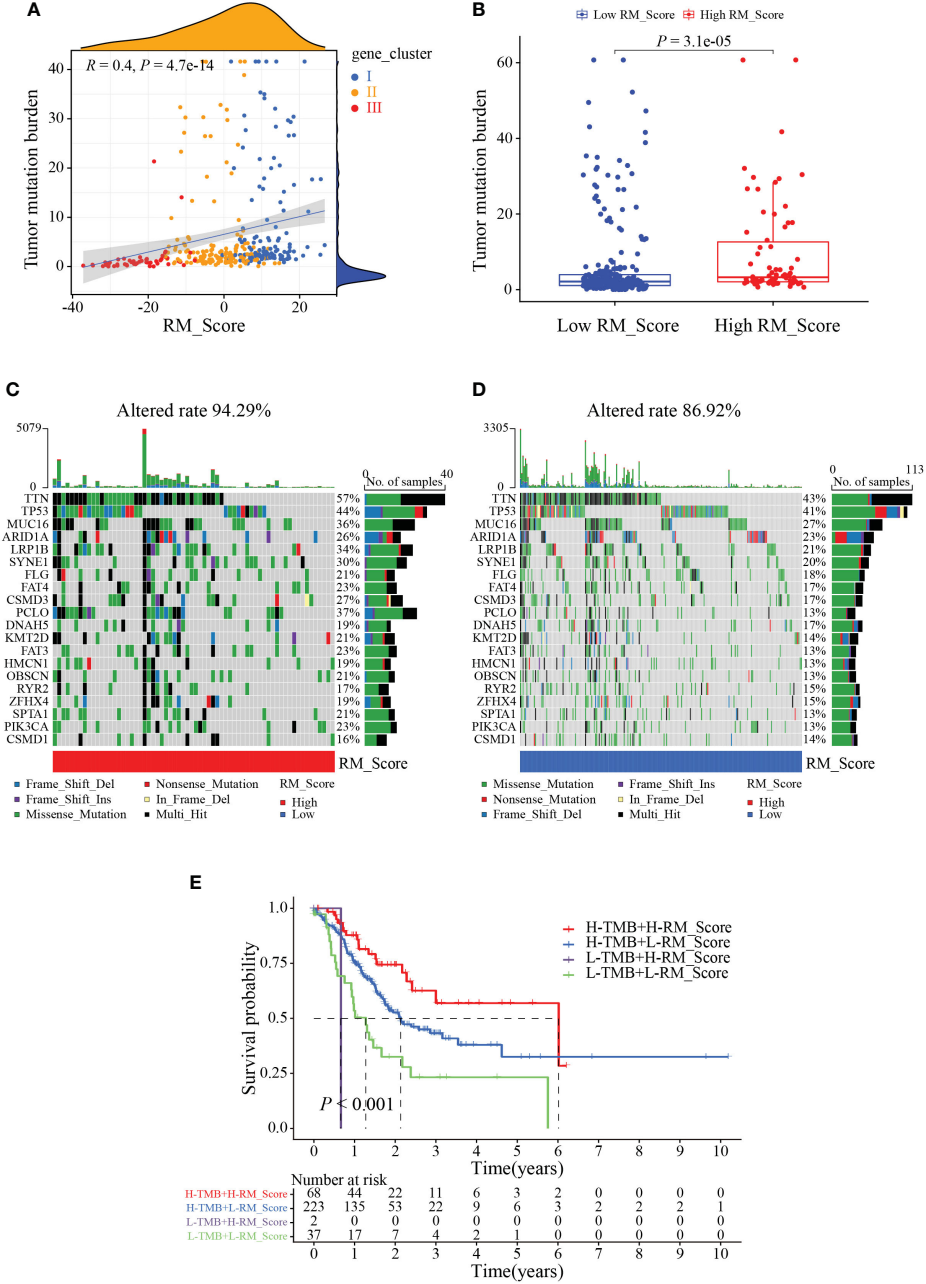
The correlation between RM_Score and TMB. **(A)** Correlations between RM_Score and TMB. **(B)** Differences in TMB between high and low RM_Score groups. **(C, D)** Waterfall plot displays the somatic mutation features that are stratified by high or low RM_Scores. **(E)** Survival analysis among four groups of gastric cancer patients according to both levels of TMB and RM_Scores.

We next investigated the application of RM_Score on the prediction of TMB, and tumor immunology. Results showed the RM_Scores were positively correlated with TMB, which was tightly associated with the response to immunotherapy (**Figures 6A, B**). The waterfall plots revealed that the mutation frequency of the high RM_Score group (94.29%) was markedly more frequent than that in the low RM_Score group (**Figures 6C, D**). Simultaneously, when the RM_Score was integrated with TMB, patients with both high RM_Score and TMB exhibited the best survival outcome (**Figure 6E**). Previous studies have unveiled RNA modification involved in a series of fundamental bioprocesses, especially in malignant immunomodulatory abnormalities (38, 39). To explore the function of RM_Score in immune regulation, the infiltration abundance of immune cells was estimated in two distinct RM_Score groups. The infiltration levels of activated mast cells and neutrophils were elevated in the high RM_Score group (**Figures S4A, B**). Simultaneously, the immune-related functions differed ubiquitously in two RM_Score groups (**Figure S4C**). Results of the relationship between RM_Score and immune checkpoints manifested PD-1 and PD-L1 expressed higher in the low RM_Score group than in the high RM_Score group, and the expression of PD-1 was negatively correlated with RM_Score (**Figures S4D–G**). These results suggested high RM_Score patients might be more sensitive to immunotherapy and benefit from it.

### 3.6 Clinical characteristics and immune subtypes based on RM_Score

To disclose the link between RM_Score and clinical characteristics and immune subtypes, correlation analysis was conducted, and we observed the grade, stage, and T stage of GC patients significantly differed in the two RM_Score groups (**Figures S5A, B**). Beyond that, to acquire whether the RM_Score impacted the immune subtypes, GC patients in both RM_Score groups were categorized into different immune subtype clusters (C1, C2, C3, and C4). The chi-square test displayed the apparent differences in immune subtypes in RM_Score groups, and the majority of GC patients were in immune subtype C2 (**Figure S5C**). Studies illustrated the immune subtype C2 dominated by IFN-γ, had a high proliferation rate and correlated with highly mutated gastric cancer largely (40). These results demonstrated that RM_Score might be a potential prognostic biomarker in evaluating clinicopathologic features and assessing the therapeutic value of immunotherapy.

### 3.7 RM_Score in the role of MSI and immunotherapy

**Figure 7:**
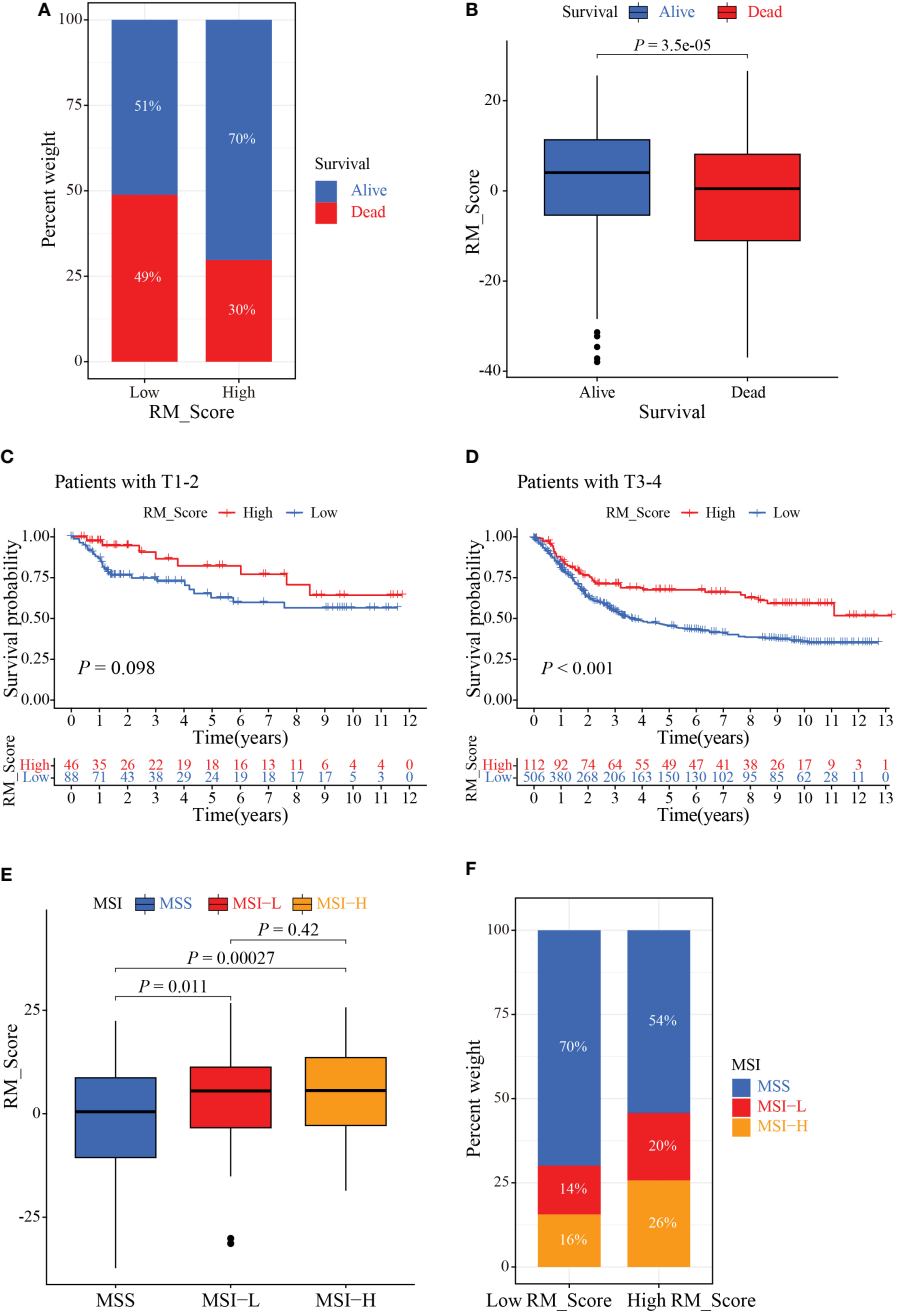
The role of RM_Score in MSI and immunotherapy. **(A, B)** Relationship between GC patients’ status and RM_Scores. **(C)** Kaplan-Meier analysis of survival rate in T1-2 cohorts of the GC patients stratified by RM_Score. **(D)** Kaplan-Meier analysis of survival rate in T3-4 cohorts of the GC patients stratified by RM_Score. **(E)** The difference of RM_Scores among three different MSI groups. **(F)** Stratified analysis of the MSI for GC patients according to RM_Score.

Since immunotherapy can improve the survival outcomes for some patients, it is crucial to identify which patients were more benefit from it. Correlation analysis revealed that patients in the high RM_Score group had impressive outcomes (**Figures 7A, B**). Through the Kaplan-Meier analysis, we found that the prognosis of GC patients was not significant in the T1-2 stages, but the high RM_Score group tended to prolong survival was observed; whereas patients with a high RM_Score had a significantly favorable survival outcome than patients with a low RM_Score in the T3-4 stages (**Figures 7C, D**). Accumulative evidence revealed that high microsatellite instability (MSI-H) patients were appropriate for immunotherapy (41, 42). Thus, a comprehensive analysis was performed to characterize the association between RM_Score and MSI. Results showed that the high RM_Score group was significantly linked with the status of MSI, which was accompanied by a better prognosis (**Figures 7E, F**), suggesting that high RM_Score group might be appropriate for immunotherapy, and the RMGs may provide a strong theoretical basis for combining tumor-targeted therapy with immunotherapy.

### 3.8 Expressional validation of three hub RMGs

**Figure 8:**
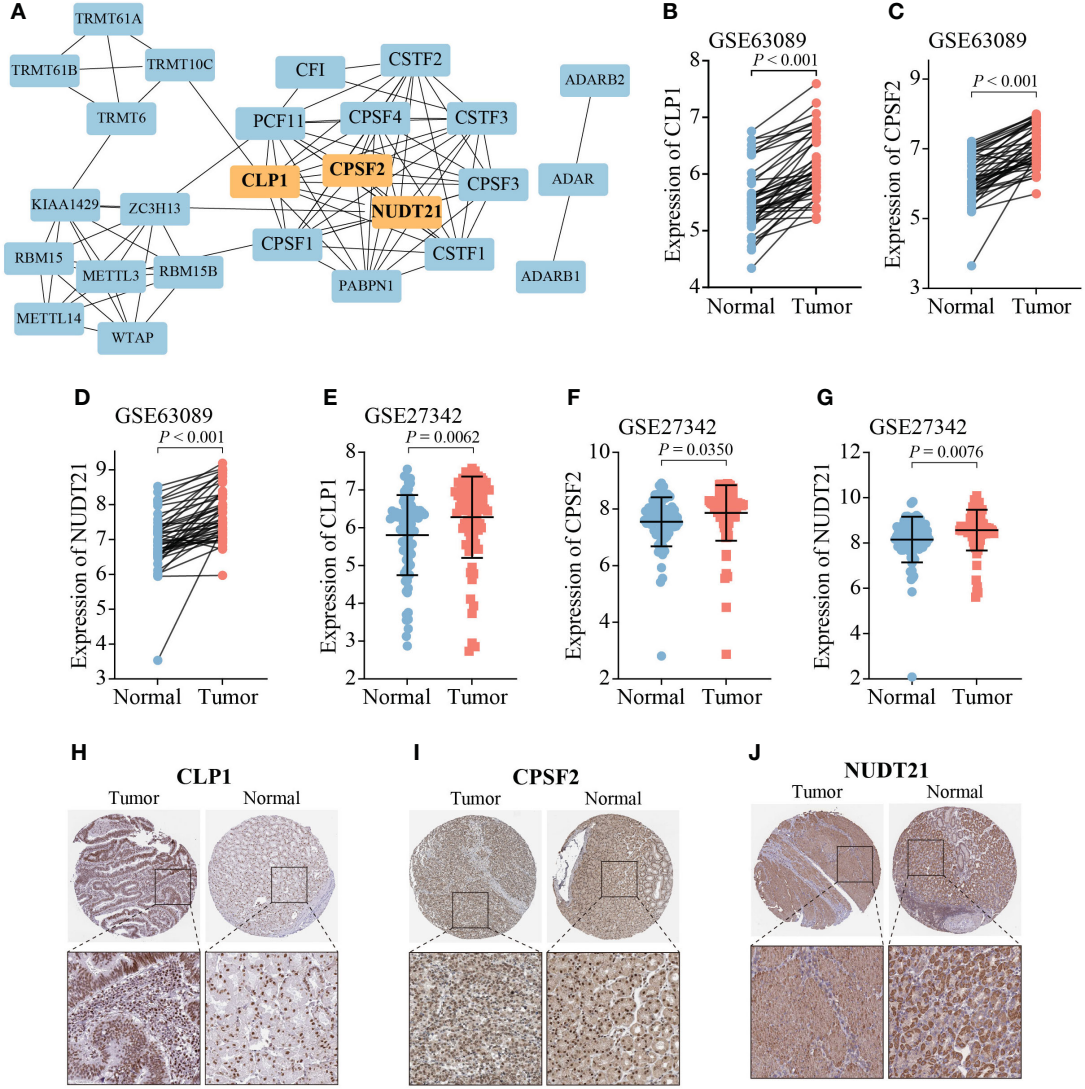
Expression analysis of three hub RMGs. **(A)** PPI network of 26 RMGs, orange modules represent the hub genes. **(B-G)** mRNA expression levels of three hub RMGs. **(H-J)** Protein expression of CLP1, CPSF2, and NUDT21.

To validate the expression of RMGs in GC, the PPI network was assembled based on the STRING database, and CLP1, CPSF2, and NUDT21 were identified as the hub genes (**Figure 8A**). We then explored the transcription and protein levels of CLP1, CPSF2, and NUDT21. Scatter plots revealed that CLP1, CPSF2, and NUDT21 expressed significantly higher in tumor tissues than in normal gastric tissues, which was completely consistent with the results of the TCGA database (**Figures 8B–G**). The immunochemistry results showed that the protein expression levels of CLP1, CPSF2, and NUDT21 were higher in GC tissues than in normal paracancerous tissues (**Figures 8H–J**).

## 4 Discussion

Accumulating studies have revealed the paramount roles of RNA modifications in innate immunity, and antineoplastic activity by interaction with various regulators, such as methyltransferases, and demethylases (43–45). Since most previous studies were confined to a single TME cell and one or two RMGs, therefore, little is known about the infiltration characteristics of TME cells mediated by integrated effects of RMGs. Recognition and determination of the role of different RNA modification patterns in the TME cell infiltration may conducive to further insights into the immune responses of the TME cell in gastric cancer.

In our study, we unveiled the genetic and transcriptional alterations of 26 RMGs and identified three distinct RNA modification patterns in gastric cancer. Compared to RM_clusters A, the expression of 26 RMGs was higher and the patients had superior clinicopathological characteristics and well prognosis in other two clusters, indicating these RMGs might potential prognostic factors for GC (46). We observed that the characteristics of the TME cell infiltration were conspicuously different among the three RM_clusters. The RM_cluster A characterized by a marked innate immune and stromal cell activation, corresponding to the immune excluded phenotype; the RM_cluster B was characterized by immunological suppression, which was consistent with the immune desert phenotype; and the RM_cluster C was represented as adaptive immune activation, corresponding to an immunologic inflammatory phenotype (47). Tumors can be divided into “hot” or “cold” tumors to reflect grading immune infiltration, and the immune desert phenotype pertains to the “cold” tumor, which lacks tumor-infiltrating lymphocytes (TILs) in the TME and can cause immunoediting and T cell escape (47, 48). The immune excluded and immune-desert phenotypes, which have massive T cell infiltration, belong to the “hot” tumors. Based on the above definitions, we observed that RM_cluster C presented a substantial stroma activation status (the ECM receptor interaction pathways), which was regarded as T cell suppressive. Thus, it is not astonishing that patients in RM_cluster C had activated innate immunity and worse survival outcomes.

As the mRNA transcriptional differences in different RNA modification patterns were closely associated with immune-related pathways. We then identified the overlapping genes based on the three RNA modification patterns. Consistent with the previous scheme, we identified three gene clusters, which were markedly linked to the activation of stroma cells and immune cells, suggesting that the RNA modification patterns involved the shaping of the TME directly. Consequently, analysis in depth of RNA modification will be instrumental in discerning the landscapes of the TME cell-infiltrating features. Considering this, we established the RM_Score system to quantify RNA modification patterns and validated its predictive ability in the clinical prognosis of GC patients. We found the RNA modification patterns characterized by immune-excluded and immune-inflamed phenotypes, displayed poor RM_Scores, whereas the RNA modification pattern that exhibited immune-excluded immunophenotype had a higher RM_Score. The RM_Score exhibited a strong positive correlation with immune cells, including CD4 T cells, CD8 T cells, and DCs, which could further explore the infiltration patterns of the TME. Additionally, GC patients with high RM_Score had a better survival outcome, indicating RM_Score was a credible tool to comprehensively evaluate the RNA modification patterns in individual tumors and also was a protective independent prognostic biomarker.

Increasing evidence revealed that patients with high TMB have a prior likelihood of immunotherapy response, particularly with PD-1/PD-L1 blockade, in tumor diagnoses based on comprehensive genomic profiling (49, 50). Our current study showed a conspicuously positive correlation between the RM_Score and TMB. Patients with low RM_Score had a lower gene mutation frequency compared with the patients with high RM_Score, and patients with both low RM_Score and low TMB showed a worse prognosis, which may be attributed to the immunosuppression caused by stromal activation (51), implying the nonnegligible role of different RNA modification patterns in the immunotherapy. We also confirmed the relationship between RM_Score and MSI status, which acts as a clinical biomarker and is correlated with immune checkpoint blockade (52, 53). Consistent with previous studies, patients with MSI-H subtypes were more sensitive to checkpoint immunotherapy, a fact that can guide rational treatment in GC (54).

In short, our study showed that the RM_Score might serve as a promising prognostic biomarker for GC patients. The RM_score could be used to determine the infiltration characterization of the TME and identify the immunophenotypes in individual tumors for clinical practice. More importantly, the RM_Score may have the potential ability to the evaluation of clinicopathological characteristics, especially in genetic variation, TMB, and MSI status. These findings provide novel assessment strategies based on RMGs for immunotherapy in GC.

## 5 Conclusions

In conclusion, our comprehensive integrated analysis of RNA modification genes revealed the effects of different RNA modification patterns on the infiltration of individual TMEs and immunotherapy in gastric cancer. The differences in RNA modification patterns may be tightly tied to the clinicopathological characteristics, immunotherapy, and prognosis of GC patients.

## Data availability statement

The original contributions presented in the study are included in the article/**Supplementary Material**. Further inquiries can be directed to the corresponding authors.

## Author contributions

Conception and design of the study: XT and JX. Provision of study material or patients: CYu, JH, PZ and YW. Collection and assembly of data: JD, YZ, XR, CYa, and GL. Analysis and interpretation of data: QJ, XK, HY, HL. Manuscript writing: All authors. Final approval of manuscript: All authors. All authors contributed to the article and approved the submitted version.

## Supplementary material

Supplementary Figure 1 The relative expression abundance of RMGs in ZC3H13 wild (blue) and ZC3H13 mutation (red) groups in GC. **(A–D)** Expression levels of m6A **(A)**, APA **(B)**, A-I **(C)**, and m1A **(D)** related genes in two distinct groups. m6A, N6-methyladenosine; APA, alternative polyadenylation; A-I, adenosine-to-inosine; m1A, N1-methyladenosine.

Supplementary Figure 2 Survival analysis of GC patients in different RMGs expression groups. **(A–D)** Survival analysis of GC patients in alternative polyadenylation modification genes expression group **(A)**, N6-methyladenosine modification genes expression group **(B)**, N1-methyladenosine modification gene expression group **(C)**, adenosine-to-inosine modification gene expression group **(D)**.

Supplementary Figure 3 Identification and analysis of DEGs subtypes in GC. **(A)** CDF curves of different numbers of consensus clusters. **(B)** The delta area of different CDF curves. **(C)** Heatmap of sample clustering; the vertical axis shows the different k values for consensus clustering. **(D–L)** Heatmaps reflect the consensus matrix for different k values.

Supplementary Figure 4 Evaluation of the immune-related functions between the two RM_Score groups. **(A, B)** Different infiltration abundance of 22 immune cells in the two RM_Score groups. **(C)** Box plot showing the differences in immune-related functions between the low and high RM_Score groups. **(D, E)** PD-L1 or PD-1 expression levels in two distinct RM_Score groups. **(F, G)** Correlations between RM_Score and PD-L1 or PD-1 expression, respectively. **P*-value < 0.05, ** *P*-value < 0.01, ****P*-value < 0.001.

Supplementary Figure 5 Identification of the clinical and immune subtypes characteristics based on RM_Score. **(A)** Distribution of clinical features between two distinct RM_Score groups in gastric cancer from the TCGA cohort. **(B)** Distribution of GC patients from the TCGA cohort between RM_Score and different clinical stages. **(C)** Differential distribution of immune subtypes characteristics in GC patients. ****P*-value < 0.001.
