# Supplementary figures and images for: A novel RNA modification prognostic signature for predicting the characteristics of the tumor microenvironment in gastric cancer
Source: Front Oncol. 2023 Feb 16;13:905139. doi: 10.3389/fonc.2023.905139 (PMC9978099; doi:10.3389/fonc.2023.905139)

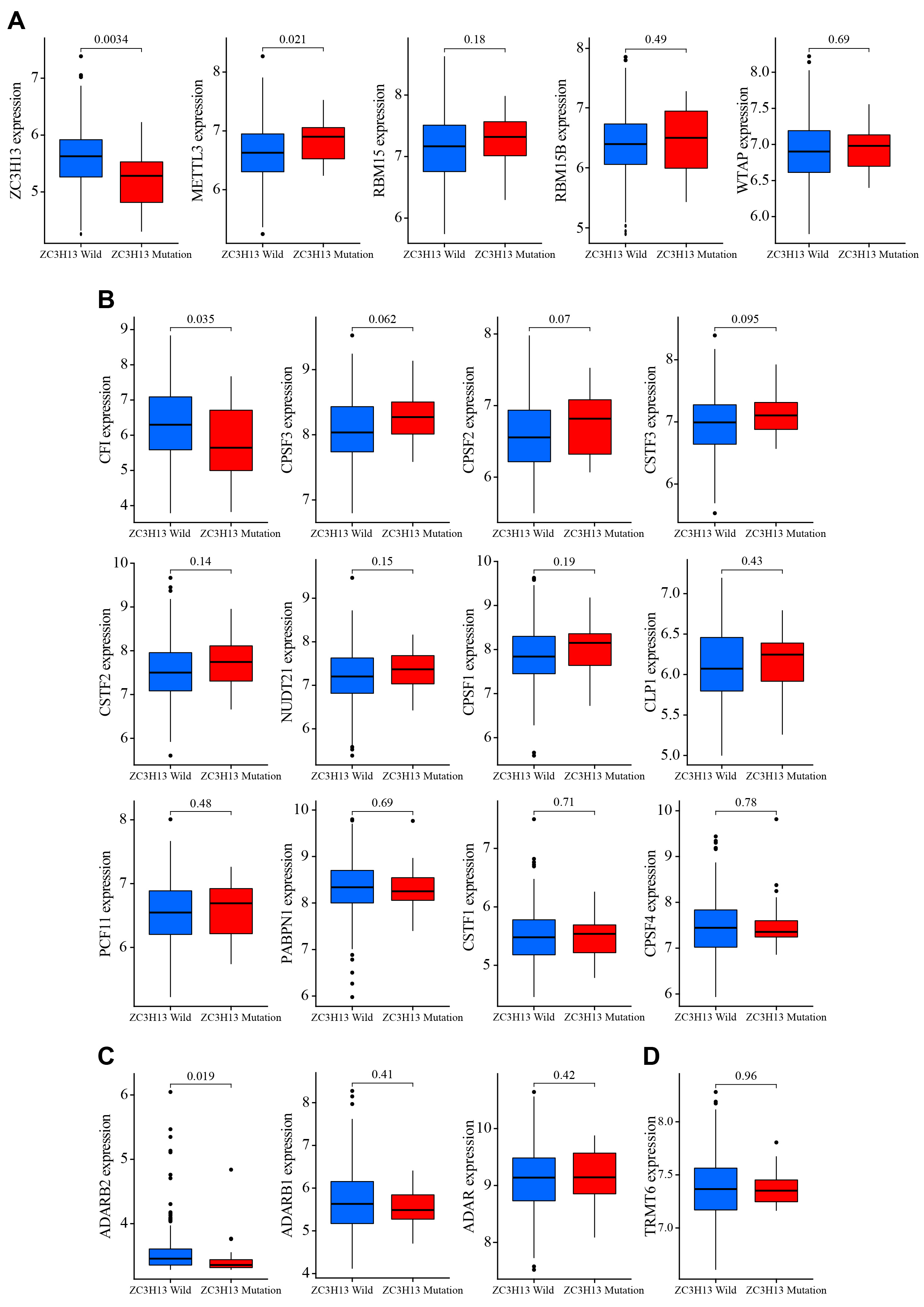

Supplement: Supplementary Figure 1 — The relative expression abundance of RMGs in ZC3H13 wild (blue) and ZC3H13 mutation (red) groups in GC. (A–D) Expression levels of m6A (A), APA (B), A-I (C), and m1A (D) related genes in two distinct groups. m6A, N6-methyladenosine; APA, alternative polyadenylation; A-I, adenosine-to-inosine; m1A, N1-methyladenosine. [file Image_1.jpeg]

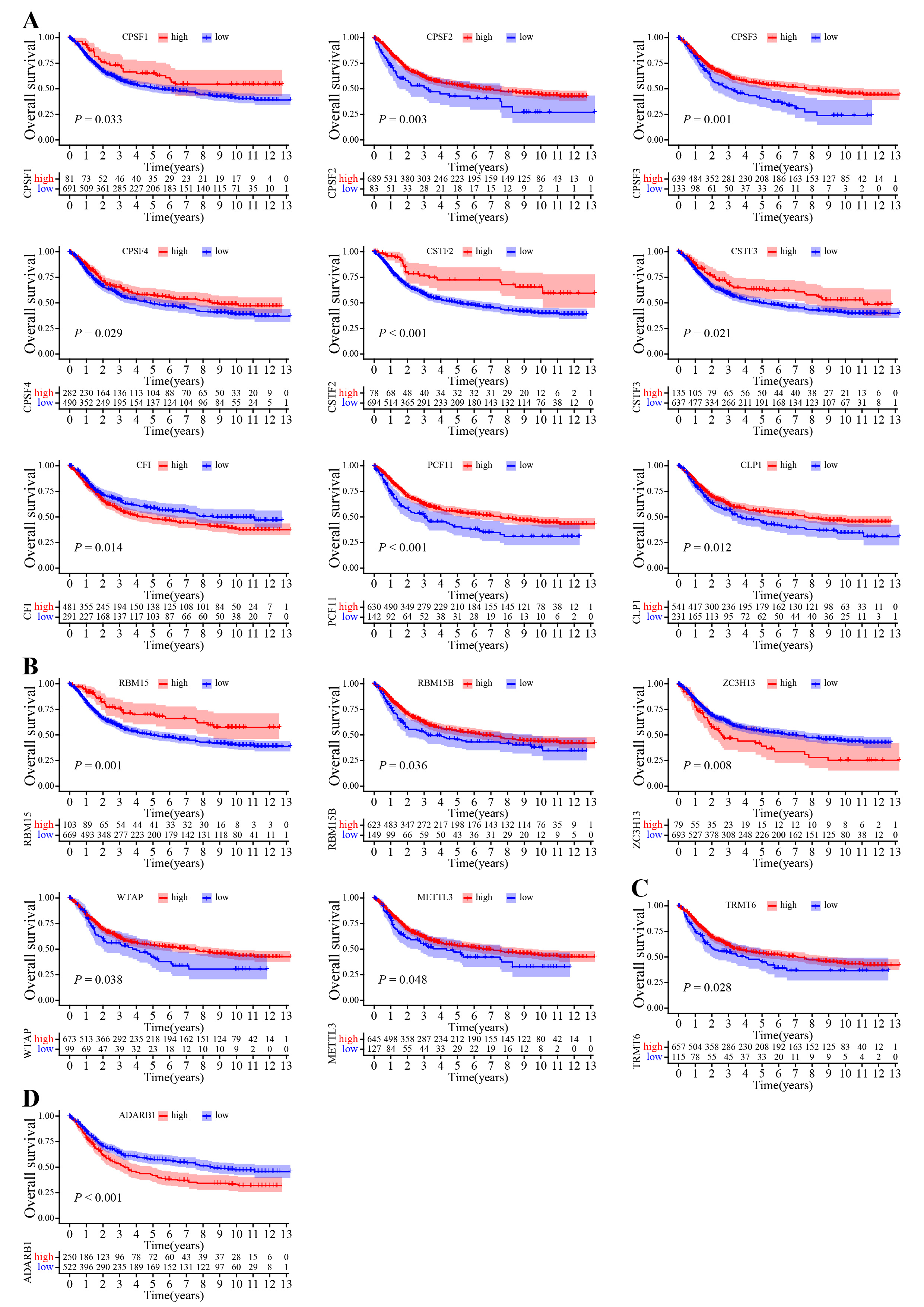

Supplement: Supplementary Figure 2 — Survival analysis of GC patients in different RMGs expression groups. (A–D) Survival analysis of GC patients in alternative polyadenylation modification genes expression group (A), N6-methyladenosine modification genes expression group (B), N1-methyladenosine modification gene expression group (C), adenosine-to-inosine modification gene expression group (D). [file Image_2.jpeg]

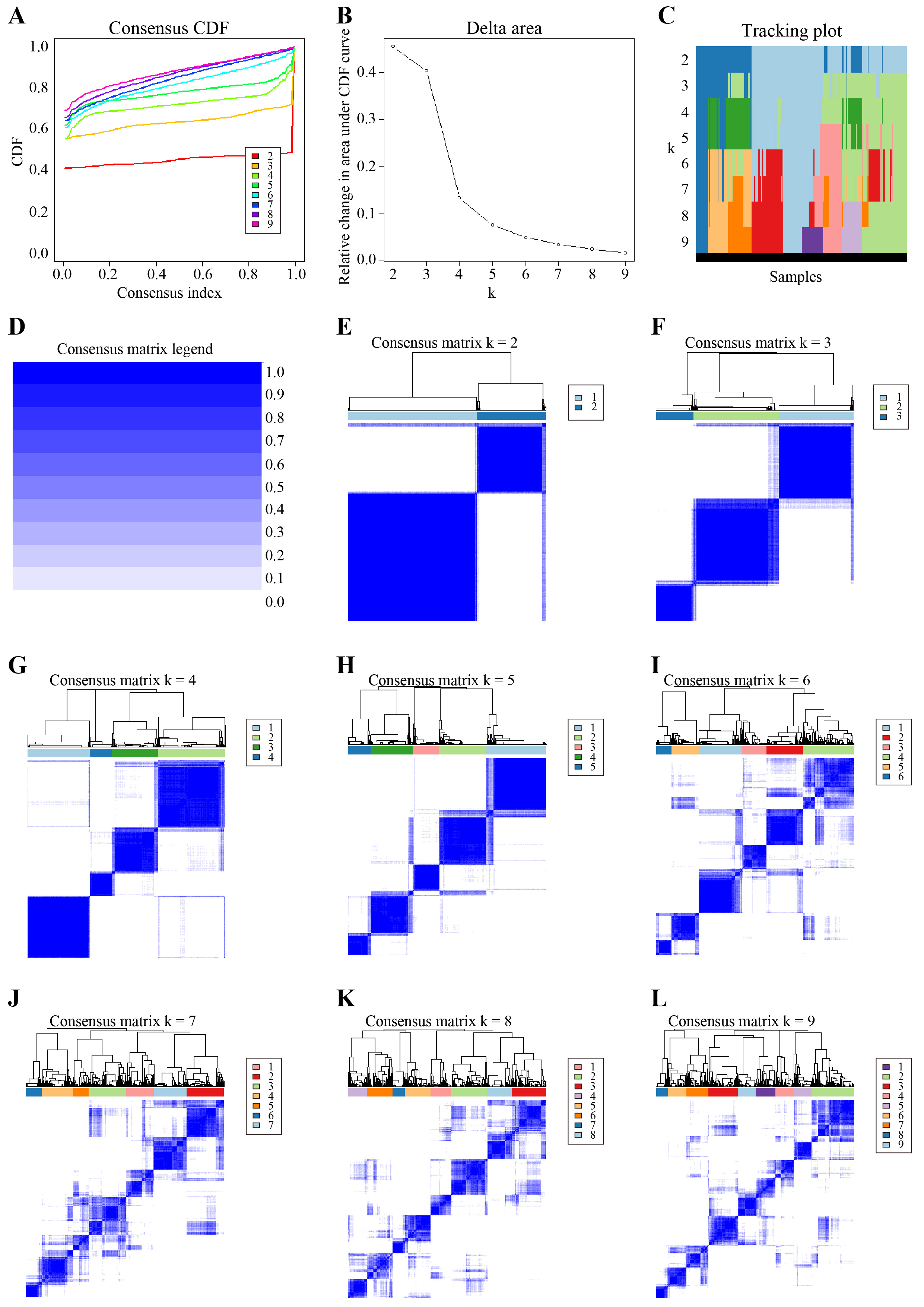

Supplement: Supplementary Figure 3 — Identification and analysis of DEGs subtypes in GC. (A) CDF curves of different numbers of consensus clusters. (B) The delta area of different CDF curves. (C) Heatmap of sample clustering; the vertical axis shows the different k values for consensus clustering. (D–L) Heatmaps reflect the consensus matrix for different k values. [file Image_3.jpeg]

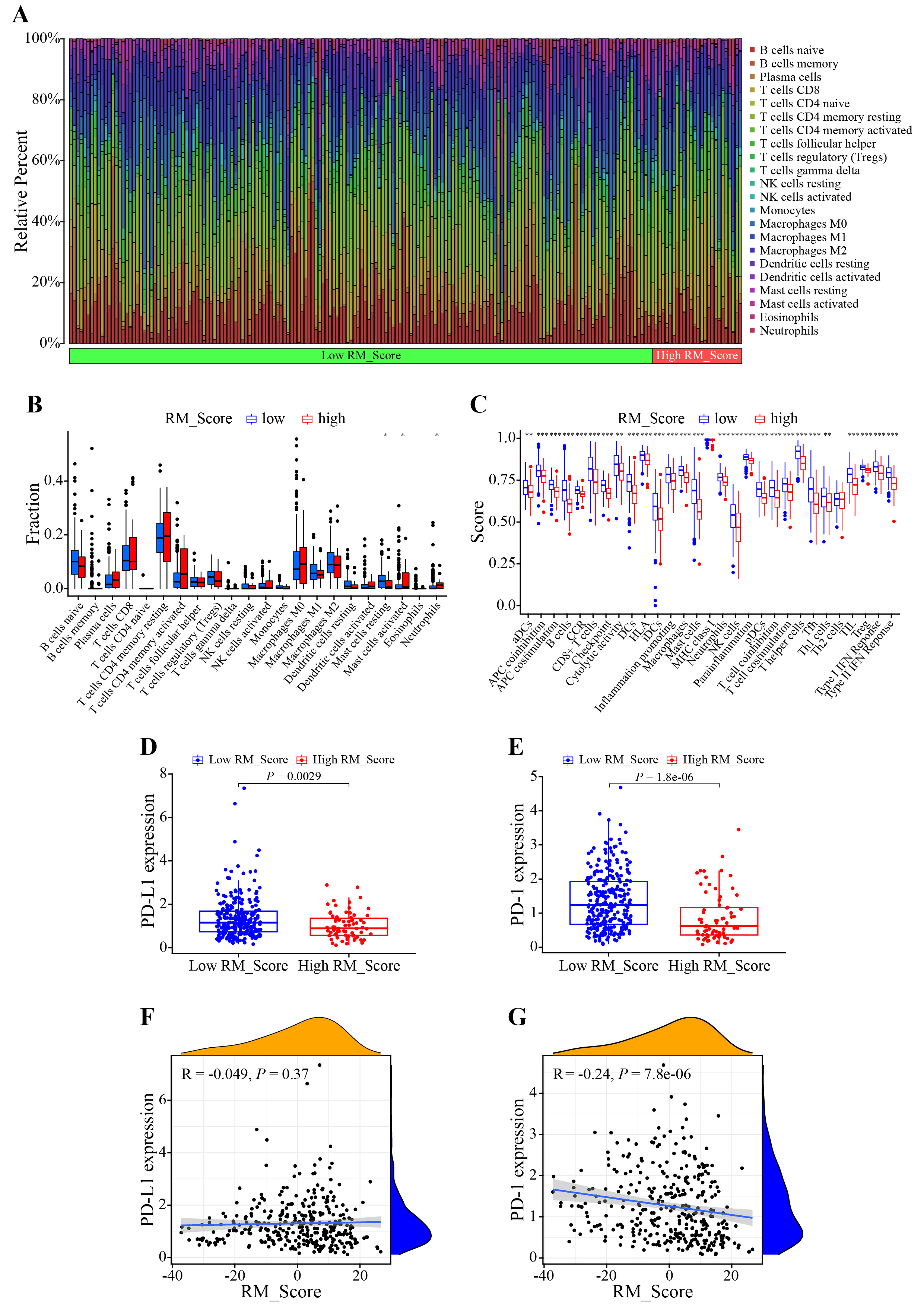

Supplement: Supplementary Figure 4 — Evaluation of the immune-related functions between the two RM_Score groups. (A, B) Different infiltration abundance of 22 immune cells in the two RM_Score groups. (C) Box plot showing the differences in immune-related functions between the low and high RM_Score groups. (D, E) PD-L1 or PD-1 expression levels in two distinct RM_Score groups. (F, G) Correlations between RM_Score and PD-L1 or PD-1 expression, respectively. *P-value < 0.05, ** P-value < 0.01, ***P-value < 0.001. [file Image_4.jpeg]

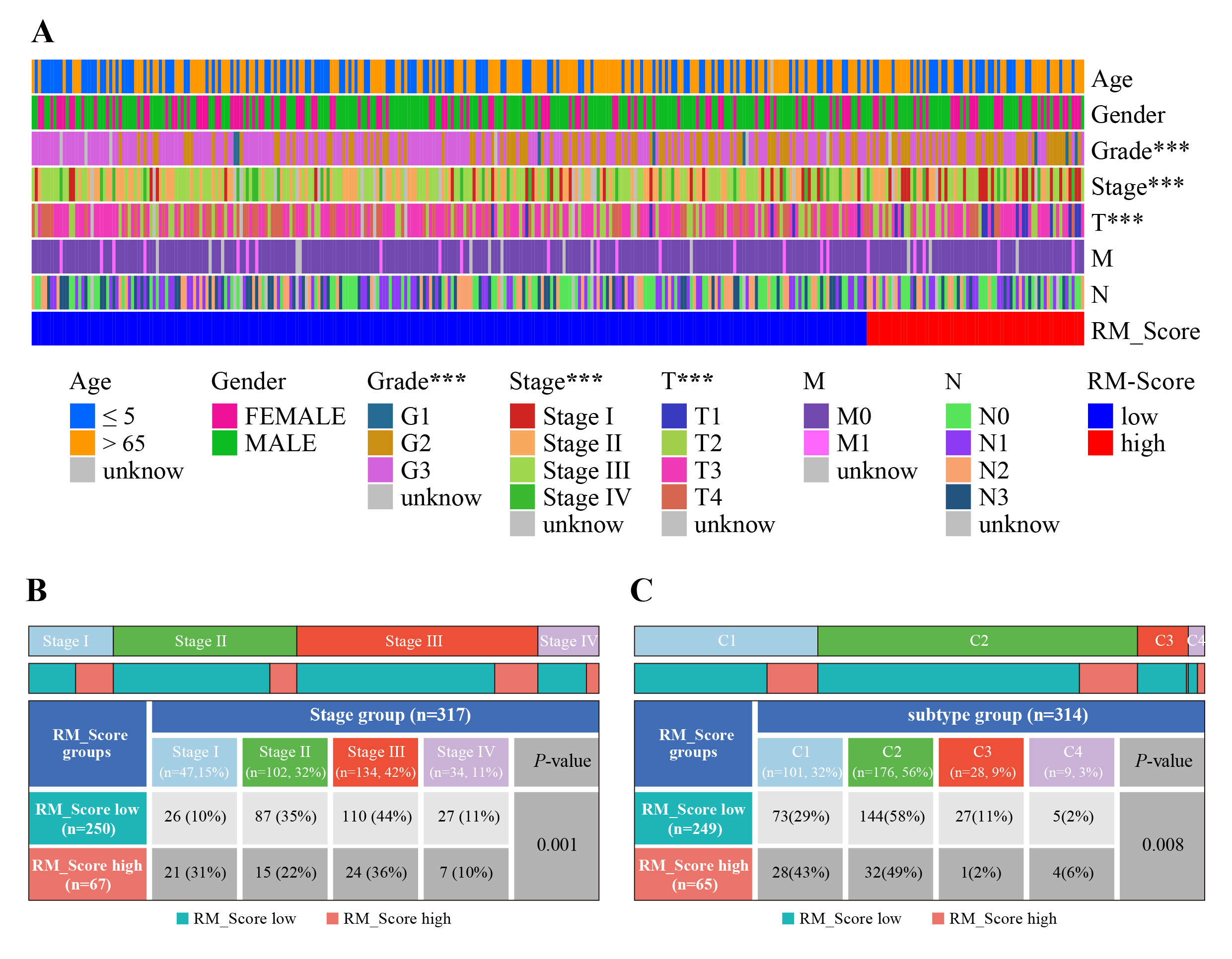

Supplement: Supplementary Figure 5 — Identification of the clinical and immune subtypes characteristics based on RM_Score. (A) Distribution of clinical features between two distinct RM_Score groups in gastric cancer from the TCGA cohort. (B) Distribution of GC patients from the TCGA cohort between RM_Score and different clinical stages. (C) Differential distribution of immune subtypes characteristics in GC patients. ***P-value < 0.001. [file Image_5.jpeg]
