# Supplementary material for: A novel RNA modification prognostic signature for predicting the characteristics of the tumor microenvironment in gastric cancer
Source: Front Oncol. 2023 Feb 16;13:905139. doi: 10.3389/fonc.2023.905139 (PMC9978099; doi:10.3389/fonc.2023.905139)
Supplement: Supplementary file 6 [file Table_1.docx]

Table S1 **|** The detailed information of 26 RMGs.

| Genes | Type of RNA adenosine modification | Regulators type |
| --- | --- | --- |
| *METTL3, METTL14, WTAP, RBM15, RBM15B, ZC3H13, VIRMA* | m^6^A | writer |
| *TRMT61A, TRMT10C, TRMT61B, TRMT6* | m^1^A | writer |
| *CPSF1, CPSF2, CPSF3, CPSF4, CSTF1, CSTF2, CSTF3, CFI, PCF11, CLP1, NUDT21, PABPN1* | APA | writer |
| *ADAR, ADARB1, ADARB2* | A-I | writer |

RMGs, RNA modification genes; m^6^A, N6-methyladenosine; m^1^A, N1-methyladenosine; APA, alternative polyadenylation; A-I, adenosine-to-inosine.
