# Supplementary material for: A novel RNA modification prognostic signature for predicting the characteristics of the tumor microenvironment in gastric cancer
Source: Front Oncol. 2023 Feb 16;13:905139. doi: 10.3389/fonc.2023.905139 (PMC9978099; doi:10.3389/fonc.2023.905139)
Supplement: Supplementary file 7 [file Table_2.docx]

Table S2 **|** The statistically significant correlations among five RMGs.

| Edge from | Edge to | Cor | *P*-value |
| --- | --- | --- | --- |
| *METTL3* | *WTAP* | 0.2940 | 6.18e-17 |
| *METTL3* | *RBM15* | 0.2477 | 2.60e-12 |
| *METTL3* | *RBM15B* | 0.3498 | 9.32e-24 |
| *METTL3* | *ZC3H13* | 0.1709 | 1.69e-06 |
| *WTAP* | *RBM15* | 0.3160 | 1.88e-19 |
| *RBM15* | *RBM15B* | 0.1581 | 9.70e-06 |
| *RBM15B* | *ZC3H13* | 0.2421 | 8.12e-12 |

RMGs, RNA modification genes.
